# Cerebral microembolism in the critically ill with acute kidney injury (COMET-AKI trial): study protocol for a randomized controlled clinical trial

**DOI:** 10.1186/s13063-018-2561-3

**Published:** 2018-03-21

**Authors:** Gabor Erdoes, Dominik E. Uehlinger, Beatrice Kobel, Monika P. Stucki, Roland Wiest, Frank Stueber, Niklaus Fankhauser, Stephan M. Jakob, Joerg C. Schefold

**Affiliations:** 1 Department of Anesthesiology and Pain Therapy, Inselspital, Bern University Hospital, University of Bern, Freiburgstrasse 18, 3010 Bern, Switzerland; 2 Department of Nephrology and Hypertension, Bern University Hospital, University of Bern, 3010 Bern, Switzerland; 3 Institute of Diagnostic and Interventional Neuroradiology, Inselspital, Bern University Hospital, University of Bern, 3010 Bern, Switzerland; 4 Clinical Trials Unit (CTU), University of Bern, 3012 Bern, Switzerland; 5 Department of Intensive Care Medicine, Bern University Hospital, University of Bern, 3010 Bern, Switzerland

**Keywords:** Renal replacement therapy, Acute kidney injury, Critically ill patients, Cerebral microembolism

## Abstract

**Background:**

Microembolism is a frequent pathological event during extracorporeal renal replacement therapy (RRT). Some previous data indicate that microemboli are generated in patients who are undergoing RRT and that these may contribute to increased cerebrovascular and neurocognitive morbidity in patients with end-stage renal disease. The current trial aims to quantify the microembolic load and respective qualitative composition that effectively reaches the intracerebral circulation in critically ill patients treated with different RRT modalities for acute kidney injury (AKI).

**Methods/design:**

The COMET-AKI trial is a prospective, randomized controlled clinical trial with a 2-day clinical assessment period and follow-up visits at 6 and 12 months. Consecutive critically ill patients with AKI on continuous renal replacement therapy (CRRT) scheduled for a switch to intermittent renal replacement therapy (IRRT) will be randomized to either switch to IRRT within the next 24 h or continued CRRT for an additional 24 h. Cerebral microembolic load will be determined at baseline, i.e., before switch (on CRRT for both groups) and on IRRT versus CRRT, whichever group they were randomized to. The primary endpoint is defined as the difference in mean total cerebral microemboli count during the measurement period on CRRT versus IRRT following randomization. Microemboli will be assessed within the RRT circuit by a 1.5-MHz ultrasound detector attached to the venous RRT tubing and cerebral microemboli will be measured in the middle cerebral artery using a 1.6-MHz robotic transcranial Doppler system with automatic classification of Doppler signals as solid or gaseous. In addition to Doppler measurements, patients will be examined by magnetic resonance imaging and neurocognitive tests to gain better understanding into the potential morphological and clinical consequences of embolization.

**Discussion:**

The results of COMET-AKI may help to gain a better insight into RRT modality-associated differences regarding microbubble generation and the cerebral microembolic burden endured by RRT recipients. Furthermore, identification of covariates of microbubble formation and distribution may help to encourage the evolution of next-generation RRT circuits including machinery and/or filters.

**Trial registration:**

ClinicalTrials.gov, ID: NCT02621749. Registered on 3 December 2015.

**Electronic supplementary material:**

The online version of this article (10.1186/s13063-018-2561-3) contains supplementary material, which is available to authorized users.

## Background

Patients undergoing renal replacement therapy (RRT) are frequently disabled, and their quality of life may be limited by dementia and silent or apparent stroke [1, 2]. Neuroimaging studies have revealed an increased prevalence of cerebral atrophy and ischemic brain lesions in RRT recipients compared to the general population following rather short periods of RRT [3–5]. In addition to traditional cerebrovascular risk factors that predispose patients undergoing RRT to increased neurological morbidity, microembolic brain injury was postulated a relevant risk factor and generation of gaseous microemboli (= microbubbles) were observed in hemodialysis circuits [6, 7]. Microemboli were also detected in arterio-venous fistulae, the subclavian vein and the carotid artery of RRT recipients. Notably, this may occur in the absence of intracardiac right-to-left shunt. Further, it is assumed that a substantial number of microemboli can evade bubble alarms in the RRT circuit, may pass the “lung barrier,” and reach the systemic arterial and cerebral circulation [8–11].

Several hypotheses exist with respect to the nature and mechanism of microemboli generation under RRT. During periods of insufficient anticoagulation on RRT circuits, the release of solid particles consisting of fibrin deposits and blood clots was postulated. More recent reports indicate that microemboli are mostly gaseous in nature and may arise either from the RRT itself (e.g., due to poor pre-dialysis de-airing, incorrect operation, malfunction of the degassing system, leakage, etc.) or appear as a result of pressure change-induced cavitation or by temperature gradients within the circuit [3, 6]. To date, the exact origin of microemboli generation and its clinical relevance are considered unclear [6].

Microbubbles from the RRT circuit travel within the bloodstream until they are trapped in the (e.g., pulmonary) microcirculation where they are slowly dissolved [6]. However, direct effects of gas bubbles in the microcirculation include ischemia and pressure changes in blood vessels and surrounding tissues [11–14]. Pressure on the endothelial cells results in increased large-pore radius and intercellular gap formation, increased hydrostatic pressure and enhanced permeability leading to interstitial edema. These effects appear potentiated by inflammatory responses and complement activation, triggering of activated polymorphonuclear leukocytes, and mast cell-derived histamine release, which results in furthermore increased vascular permeability. Activation of endothelial cells and platelets further results in activation of coagulation cascades and clot formation in direct proximity to the bubble. This may ultimately cause a (permanent) elevation of pulmonary artery pressures via increased pulmonary vascular resistance for example.

In the intensive care unit (ICU), RRT is typically provided by a continuous (CRRT) or an intermittent (IRRT) modality. Both modalities are considered equivalent in terms of clinical outcome and mortality [15–18]. A key difference between respective modalities is application of higher blood flow rates in IRRT (i.e., typical blood flow rates of 200–300 ml/min) compared to CRRT (i.e., typical blood flow rates: 100–150 ml/min). Thus, IRRT aims at higher urea solute clearance within a shorter treatment period (typically 4 h of IRRT applied).

The aim of the COMET-AKI trial is to investigate the impact of CRRT and IRRT on microbubble/cerebral microemboli generation in a cohort of critically ill patients with dialysis-dependent acute kidney injury (AKI). Our hypothesis is that microbubble generation hemodialysis circuits and the subsequent number of cerebral microemboli is related to the blood flow rate and associated arterial and venous pressures. As IRRT is associated with higher blood flow rates and increased dynamic pressure gradients, IRRT may theoretically generate higher microbubble rates and thus a larger cerebral microembolic burden.

## Methods/design

The cerebral microembolism in the critically ill with acute kidney injury (COMET-AKI) trial is a prospective, randomized controlled clinical trial with a 2-day assessment period and follow-up visits at 6 and 12 months. The primary objective is to investigate the impact of CRRT and IRRT on microbubble-/-cerebral microemboli generation in a cohort of critically ill patients with dialysis-dependent acute kidney injury.

The Cantonal Ethics Committee (CEC) has approved the study protocol (KEK No. 199/15, version 3.0, December 2015). The trial is registered at ClinicalTrials.gov (NCT02621749). The study will be performed in accordance with the study protocol and with principles enunciated in the current version of the Declaration of Helsinki, the guidelines of Good Clinical Practice (GCP) issued by the International Council on Harmonization (ICH), the European Directive on medical devices 93/42/EEC and the ISO Norm 14,155 and ISO 14971, and applicable Swiss Law. COMET-AKI is monitored by the Department of Anesthesiology and Pain Therapy of the University of Bern. The study protocol follows the Consolidated Standards of Reporting Trials (CONSORT) guidelines. All relevant data will be stored on an in-house research platform, which is a dedicated server for research data management.

### Study population and patient allocation

**Fig. 1:**
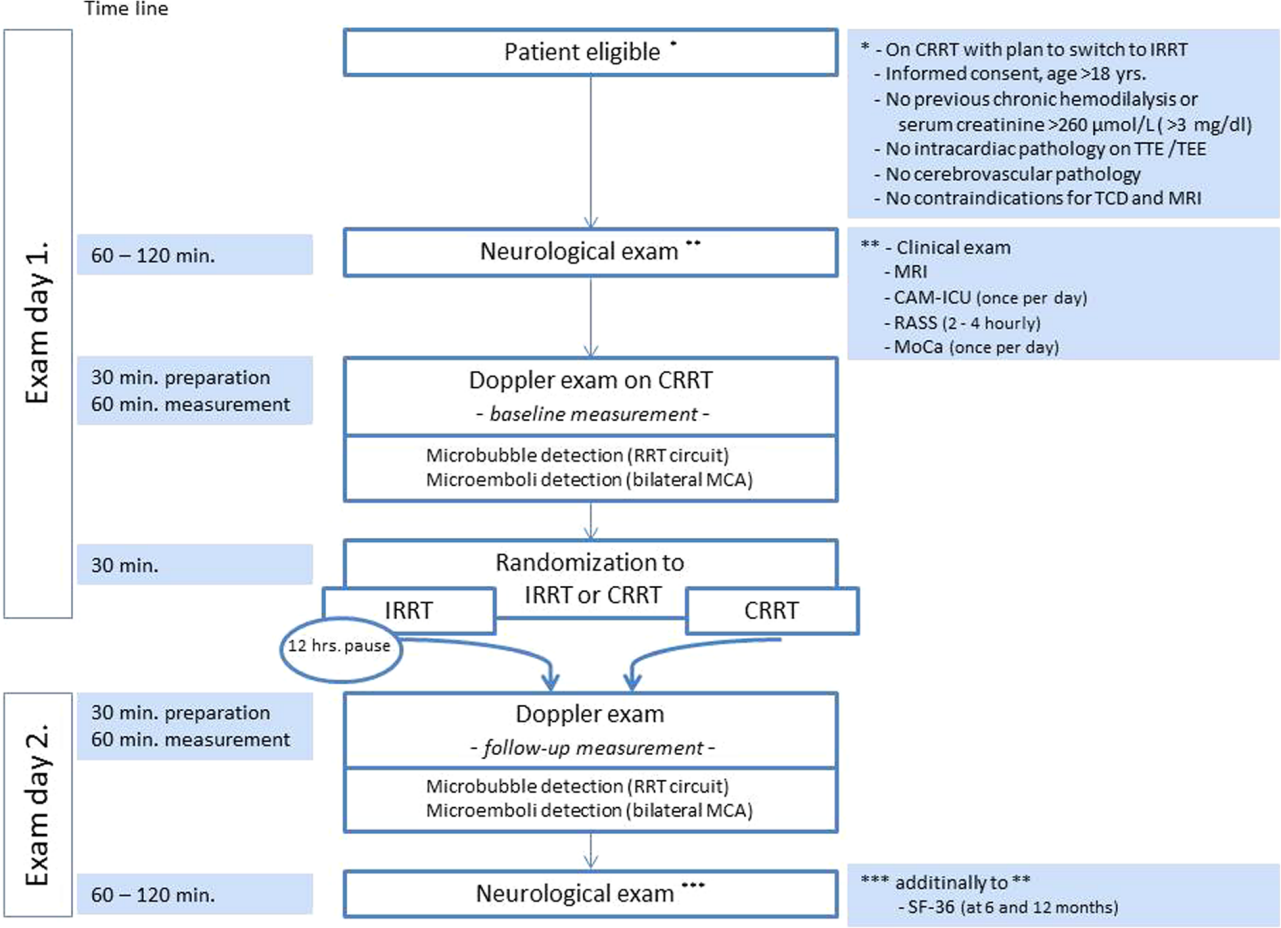
Patient flow diagram with allocation to CRRT versus IRRT. *CRRT* continuous renal replacement therapy, *IRRT* intermittent renal replacement therapy, *RRT* renal replacement therapy, *TTE* transthoracic echocardiography examination, *TEE* transesophageal echocardiography examination, *TCD* transcranial Doppler ultrasound, *MRI* magnetic resonance imaging, *CAM-ICU* Confusion Assessment Method for the Intensive Care Unit, *RASS* Richmond Agitation and Sedation Scale, *MoCA* Montreal Cognitive Assessment, *MCA* middle cerebral artery, *SF-36* Short Form (36) Health Survey

**Fig. 2:**
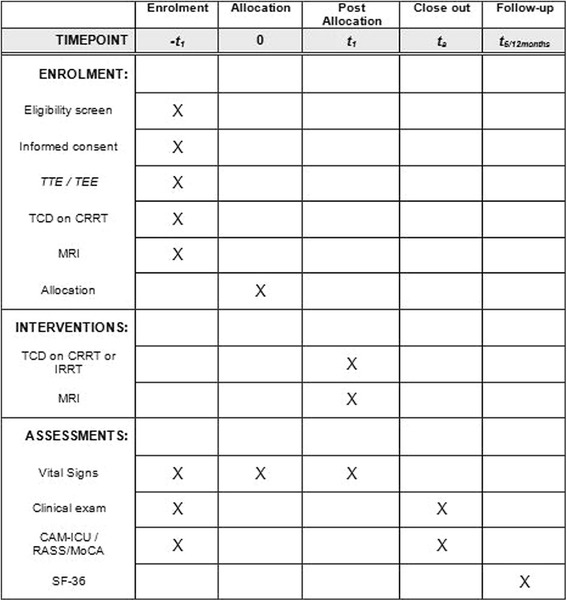
Standard Protocol Items: Recommendations for Interventional Trials (SPIRIT) Figure: schedule of enrollment, interventions and assessments for the COMET-AKI trial. *CRRT* continuous renal replacement therapy, *IRRT* intermittent renal replacement therapy, *TTE* transthoracic echocardiography examination, *TEE* transesophageal echocardiography examination, *TCD* transcranial Doppler ultrasound, *MRI* magnetic resonance imaging, *CAM-ICU* Confusion Assessment Method for the Intensive Care Unit, *RASS* Richmond Agitation and Sedation Scale, *MoCA* Montreal Cognitive Assessment, *MCA* middle cerebral artery, *SF-36* Short Form (36) Health Survey

Consecutive critically ill patients treated with CRRT for AKI, and scheduled to be switched (as determined by the treating ICU physician in charge) to IRRT, will be randomized by an independent computer-based procedure to either switch to IRRT the next day or to continued CRRT for one additional day. Doppler measurements will be performed during CRRT for both groups before the switch (baseline, examination day 1) and after the switch (follow-up measurement, examination day 2) in the CRRT and IRRT groups, respectively. In the IRRT group, there will be a RRT pause of 12 h between CRRT termination and IRRT start in order to allow equilibration and wash out of potential CRRT-induced microemboli. Transcranial Doppler (TCD) detection of cerebral microemboli will be performed bilaterally and continuously in both middle cerebral arteries (MCAs) for 30 min. Additionally, microbubble generation will be simultaneously recorded in the RRT circuit by extracorporeal Doppler detector in the venous tubing, placed 10 cm downstream of the air detector (Fig. 1). No interventions (e.g., bolus intravenously administered (IV) drug injections, line flushing, change of infusion rate, blood sampling or other manipulations) will be allowed during Doppler examinations. At the beginning and at the end of the study period, patients will undergo routine clinical examinations to evaluate for neurological and neurocognitive disturbances. A magnetic resonance imaging (MRI) examination will be performed after inclusion of the patient in the study and again after completion of the follow-up Doppler measurements (Fig. 2).

### Consent procedure

The investigators will explain to each participant the nature of the study, its purpose, the procedures involved, the expected duration, the potential risks/benefits or any potential discomfort. Each participant will be informed that the participation in the study is voluntary and that they may withdraw from the study at any time and that withdrawal of consent will not affect their subsequent medical assistance and/or treatment. All participants for the study will be provided with a Participant Information Sheet and a Consent Form describing the study and providing sufficient information for the participant to make an informed decision about their participation in the study. The formal consent of a participant/next of kin, using the approved Consent Form, will be obtained before the participant is submitted to any study-specific procedure. The participant/next of kin must read and sign the informed consent and will be given a copy of the signed document. In case of inability to give consent, the patient’s legal representative will be asked for the patient’s participation in the study. In case of deferred consent and as soon as the patient is able to give consent, the patient will be informed in detail about the study. Patients may withdraw from the study at any stage by oral or written declaration. In case of withdrawal of informed consent (or in case of non-compliance, disease progression, safety concerns) all study relevant data will be anonymized following data base closure.

### Study periods and visits

The study includes a 2-day assessment period/clinical phase and follow-up visits at 6 and 12 months. During the clinical phase, participants will attend five study visits. The overall duration of the clinical phase will be approximately 207 min for each participant.

Procedures at each visit include (Table 1):

**Table 1:** Study measures at each visit

	Visit 1: screening	Visit 2: baseline neurocognitive examinations	Visit 3: Doppler measurement CRRT	Visit 4: randomization	Visit 5: Doppler measurement IRRT or CRRT	Visit 6: follow-up neurocognitive examinations
Duration of visit	30 min	60–120 min	90 min	30 min		60–120 min
Recruitment	X					
Consent Form	X					
Inclusion/exclusion	X					
Medical history	X					
TTE/TEE	X					
Assess vital signs		X	X	X	X	X
External randomization				X		
Clinical examination		X				X
Doppler examination			X		X	
MRI		X				X
CAM-ICU		X				X
RASS		X				X
MoCA		X				X
SF-36^a^						X

^a^After 6 and 12 months. *CAM-ICU* Confusion Assessment Method for the Intensive Care Unit, *CRRT* continuous renal replacement therapy, *IRRT* intermittent renal replacement therapy, *MoCA* Montreal Cognitive Assessment, *SF-36* Short Form (36) Health Survey, *MRI,* magnetic resonance imaging, *RASS* Richmond Agitation and Sedation Scale, *TEE* transesophageal echocardiography, *TTE* transthoracic echocardiography

- Visit 1: Recruitment and screening
  - Patients/next of kin are recruited on site. If the treating intensivist identifies a patient whose clinical status allows switching from CRRT to IRRT, has no exclusion criteria (especially, no pathology on TTE/TEE examination) and fulfills the inclusion criteria, they will inform the patient about the study together with a study representative and will hand out the Patient Information Sheet and Consent Form (in case of inability to consent: the legal representative/next of kin will be informed). The treating intensivist then passes on this information to the principal investigator (PI) or his designee. If the patient (or the legal representative) is interested in participating, the PI will then contact the patient during his visit on the ICU (in case of inability to consent: the legal representative will be reached) to inform in detail about the study and to obtain written informed consent.
- Visit 2: Baseline neurocognitive examinations
  - The PI (or his designee) performs the clinical examination and the neurocognitive tests.
- Visit 3: Doppler measurements on CRRT
  - The PI (or his designee) performs the Doppler measurements during CRRT therapy.
- Visit 4: Randomization
  - At the end of examination day 1, the PI (or his designee) send out a request for patient randomization to the external institution performing the computer-based randomization (CRRT versus IRRT groups).
- Visit 5: Doppler measurements on CRRT
  - The PI (or his designee) performs the Doppler measurements during CRRT or IRRT.
- Visit 6: Follow-up neurocognitive examinations
  - The PI (or his designee) performs the clinical examination and the neurocognitive tests. After 6 and 12 months, the SF-36 Health Survey is performed by the PI (or his designee) by telephone.

### Eligibility criteria

Participants fulfilling all of the following inclusion criteria are eligible for the study:

- Age aged over 18 years
- Written informed consent as documented by the signature of the patient or their legal representative (in case of inability to give consent).

The following exclusion criteria apply:

- Presence of atrial septal defect (intracardiac shunting), severe aortic/mitral valve pathology or valvular endocarditis in routine transthoracic (TTE) or transesophageal echocardiography (TEE) examination (before baseline Doppler measurement)
- Presence of chronic atrial fibrillation or other irregular cardiac rhythm
- Known cerebrovascular pathology (carotid and/or vertebral artery stenosis > 70%, cerebrovascular insult)
- Contraindications for MRI (e.g., pacemaker, hemodynamic instability precluding patient transfer to MRI)
- Missing bilateral temporal window for transcranial Doppler ultrasound (TCD) examination
- Allergy to any plastic contained in the investigative devices (i.e., head frame, TCD probe) or to ultrasound gel
- Patients with chronic kidney disease (serum creatinine > 3 mg/dl) and/or patients undergoing chronic hemodialysis therapy
- Pregnant or lactating patients
- Patients institutionalized for neurological, psychiatric, and/or neurocognitive reasons
- Inability to follow the procedures of the study, e.g., due to language problems, psychological disorders, dementia, etc. of the participant
- Participation in another study with an investigational drug within the 30 days preceding and during the present study
- Recent hospitalization on ICU with RRT requirement
- Previous enrollment into the current study

### Sample size calculation

Based on our preliminary study, [19] we expect a true difference in the mean high-intensity transient signals (HITS) value of 700 and a standard deviation of 950 HITS between the two groups of interest. With a desired power of 0.8 and an alpha of 0.05, the number of required patients is 58 (29/group). Taking into consideration a dropout rate of 15%, we plan to include a total number of patients of 66.

### Study endpoints and statistical analyses

The primary endpoint is defined as the difference in mean total cerebral microemboli count during the measurement period on CRRT versus IRRT following randomization.

Secondary endpoints include:

1. Increase in cerebral microemboli count and rate (= count/RRT minute) in the middle cerebral artery in CRRT (CRRT group) versus IRRT (IRRT group) in relation to the baseline measurement in both groups during CRRT
2. Increase in microbubble count and rate (= count/RRT minute) in the venous outflow of the RRT circuit in CRRT (CRRT group) versus IRRT (IRRT group) in relation to the baseline measurement in both groups during CRRT
3. Relationship between the counts/rates of microbubbles and cerebral microemboli
4. Volume and number of new ischemic lesions on periprocedural MRI and their correlation to cerebral microembolic load/microbubble count
5. ICU mortality, in-hospital and 30-day mortality, intensive care unit length of stay, total hospital length of stay, days on RRT
6. Relation of the findings to underlying reason for AKI/need for RRT (descriptive analysis)
7. Relation of the findings with regard to hemodynamic data, if available (heart rate, systolic blood pressure, diastolic blood pressure, central venous pressure, vasopressor usage)

The primary endpoint results from the difference in mean total cerebral microemboli count in CRRT versus IRRT recipients compared with analysis of covariance (ANCOVA). To assess relationships between cerebral microemboli/microbubble count and the number of ischemic lesions on MRI, the Pearson correlation coefficient will be used. Other parameters will be analyzed in a descriptive fashion with parametric or non-parametric statistical methods. A significance level of 0.05 will be considered.

### Study devices and neurocognitive examinations

1. Renal replacement therapy
  Continuous renal replacement therapy is provided by PrismaflexTM (Gambro Hospal GmbH, Danziger Str. 23, 81,194 Gröbenzell, Germany). Intermittent renal replacement therapy is provided by Dialog + (B Braun Melsungen, AG, Carl-Braun-Strasse 1, 34,212 Melsungen, Germany). CRRT will be performed as follows: continuous veno-venous hemodialysis and filtration (CVVHDF) applied daily for 24 h (target) using a poly-sulfone synthetic membrane and bicarbonate-buffered substitution fluids (blood flow 150 ml/min, dialysate flow 1000 ml/h, substitute 500 ml/h, if applicable: pre-pump substitute containing citrate 1500 ml/h). Regional citrate anticoagulation (at a citrate dose of 3 mmol/l) will be used as standard anticoagulant. A delivered CRRT “dose” of > 30 ml/kg body weight/h applies. IRRT will be performed for 4 h using volumetric ultrafiltration control, water purified by reverse osmosis and bicarbonate-buffered dialysate. Blood flow and dialysate flow will be kept at 300 ml/min and 500 ml/min, respectively
2. Doppler measurements
  Cerebral microemboli detection will be performed with TCD (Delica 9UA series, Delica, Shenzen, China), which is equipped with a 1.6-MHz robotic transducer and a commercially available computer. The TCD probes will be mounted bilaterally over the temporal bone window using a dedicated size-adjustable probe-holder. HITS will be detected using the current version of EDS software (SMT medical, Wurzburg, Germany). TCD measurements will be performed according to the guidelines established by the International Consensus Group on Microembolus Detection. The MCA will be automatically insonated by the robotic TCD probes, and the Doppler velocity spectral range will be adapted to the expected maximum MCA flow velocity. Power output and gain settings will be adjusted to provide an optimal signal-to-noise ratio. On the basis of neural network technology, the EDS software automatically classifies every intensity increase within the Doppler spectrum as a cerebral microembolus (solid or gaseous) or as an artefact. After microemboli detection is complete, two experts, blinded to the study groups, will review all recordings off-line in order to confirm correct signal detection and differentiation. Microbubble detection in the RRT circuit is performed using the CMD-20 1.5-MHz ultrasound detector (Hatteland Instrumentering, Røyken, Norway) connected to the venous tubing and placed 10 cm downstream to the air detector. The device is a microbubble detector specifically designed for extracorporeal lines with an operation principle based on the pulsed Doppler technique; when the ultrasonic pulse hits a microbubble in the extracorporeal line the pulse will be partially reflected to the probe but with a different frequency than the transmitted wave (Doppler shift). The amplitude of the Doppler shift is proportional to microbubble diameter which allows for the possibility to classify both the size and the count of each microbubble passing the tube
3. Magnetic resonance imaging
  Imaging studies are performed on either a 1.5-T or a 3-T Siemens MRI system (Magnetom Avanto, Magnetom Trio; Siemens Medical Solution, Erlangen, Germany) with a 12-channel coil.
  The MRI protocol encompasses a routine MRI protocol with following sequences: axial diffusion-weighted imaging (DWI), axial fluid-attenuated inversion recovery (FLAIR), susceptibility-weighted imaging (SWI), time-of-flight (ToF) angiography and axial T1wSE post contrast
4. Neurocognitive examination
  To access neurocognitive and health disturbances, patients are subjected to a battery of tests: (1) Confusion Assessment Method for the Intensive Care Unit (CAM-ICU), (2) Richmond Agitation and Sedation Scale (RASS), (3) Montreal Cognitive Assessment (MoCA) and (4) the Short Form (36) Health Survey (SF-36)

### Severe adverse events and safety

Severe adverse events are assessed by the PI and are Medical Dictionary for Regulatory Activities (MedDRA)-coded in the Case Report Form (CRF) during the clinical phase and the follow-up period. All adverse events will be reported to the CEC in accordance with all national and institutional requirements.

### Quality assurance and control

Study data are recorded manually on the paper CRF (p-CRF) and entered after completion of the measurements in each participant into an electronic data base (double data entry). For each enrolled study participant, a CRF is maintained. CRFs are kept current to reflect subject status at each phase during the course of study. Participants cannot be identified in the CRF by name, initials and/or birth date. Appropriate coded identification with participant number in combination with date of the investigation (year, month, day) is used. Authorization for CRF entries is provided on the form “*Staff education, roles, responsibilities and signatures*” submitted as a unique document to the CEC.

### Specification of source documents

Source data include original documents relating to the study, as well as the medical treatment and medical history of the participant. All documents containing measurements (e.g., CRF) or other patient-related data (e.g. demographic data, visit dates, participation in study and Informed Consent Forms, randomization number, serious adverse events, and concomitant medication, results of relevant examinations) are considered source documents.

### Record keeping/archiving

All study data will be archived for a minimum of 10 years after study termination or premature termination of the clinical trial.

### Data management

At the departmental research platform, three locally separated dedicated servers are installed for research data management. The servers are running on Windows Server and using SQL Server (Microsoft Corporation, One Microsoft Way, Redmond, WA 98052-7329, USA) for the relational data base. The web application LabKey Server is implemented in Java and is running on the Apache Tomcat web server. The data are stored in relational data base engines such as PostgreSQL or Microsoft SQL server.

### Data security, access and backup

LabKey Server provides web application security and can be configured for Secure Socket Layer (SSL)-encrypted data transfer. The application has a group and role-based security model. Each user belongs to one or more security groups with specific sets of permissions in relation to folder or projects in the system. Only dedicated site administrators have access to the administration console enabling user management and changing security settings. Data are stored and visualized in data grids either in the format of datasets, lists or assays. Each change of data is tracked and documented in corresponding audit log files. The system can only be accessed entering a user name and password. All events are recorded in the user event list of the audit log files.

The application is running on a productive server with an image on a backup server. In addition, a regularly backup of the whole data base is implemented using storage servers.

### Analysis and archiving

LabKey Server provides data analysis by integrated tools for creating filtered views and charts. All data can be exported in different formats (Excel, text, queries) suitable for transfer to a statistical software package of choice. In addition, data can be analyzed creating R views. All data will be archived and secured in the data base as long as required by legislation.

### Electronic and central data validation

Data validity will be controlled by cross-checking the p-CRFs and the electronic data bases.

### Dissemination policy

We plan to publish the data in a peer-reviewed, high-ranking clinical journal.

## Discussion

The cerebral microembolic burden during RRT may represent an underestimated problem that can contribute to neurological complications and adverse clinical outcomes. Associated repeated hospital stays and outpatient follow-ups can be considered potential additional factors leading to an increase in healthcare costs. We expect that COMET-AKI provides more detailed insight in RRT modality-associated differences with regard to microbubble generation and cerebral microembolic burden.

Currently, it seems generally accepted by physicians and manufacturers that microbubbles are generated within the RRT circuit and that some bubbles may enter the patient’s cerebral circulation during RRT sessions. With no apparent immediate clinical side effects for RRT-treated patients, the problem of microbubbles in RRT patients appears currently not within the scope of interest. If a significant number of microbubbles would indeed be detected within the systemic circulation of the patient (i.e., in the cerebral vessels), the potential problem of microbubbles in RRT recipients may become even more significant. Our data may further provide the scientific basis for longitudinal studies relating cerebral microbubbles to changes in cognitive functions. Furthermore, identification of covariates of bubble formation and distribution may help to encourage the evolution of next-generation RRT circuits, machinery, and/or filters with reduced microbubble generation and distribution (Additional file 1).

### Trial status

Recruitment started in October 2016 and is estimated to be completed in October 2018.

## Additional file

Additional file 1: SPIRIT 2013 Checklist: recommended items to address in a clinical trial protocol and related documents. (DOC 122 kb)

## Abbreviations

AKI: Acute kidney injury
ANCOVA: Analysis of covariance
APACHE: Acute Physiology And Chronic Health Evaluation
CAM-ICU: Confusion Assessment Method for the Intensive Care Unit
CEC: Cantonal Ethics Committee
CONSORT: Consolidated Standards of Reporting Trials
CRF: Case Report Form
CRRT: Continuous renal replacement therapy
CVVHDF: Continuous veno-venous hemodialysis and filtration
DWI: Diffusion-weighted imaging
FLAIR: Fluid-attenuated inversion recovery
GCP: Good Clinical Practice
HITS: High-intensity transient signals
ICH: International Council on Harmonization
ICU: Intensive care unit
IRRT: Intermittent renal replacement therapy
MCA: Middle cerebral artery
MedDRA: Medical Dictionary for Regulatory Activities
MoCA: Montreal Cognitive Assessment
MRI: Magnetic resonance imaging
PI: Principal investigator
RASS: Richmond Agitation and Sedation Scale
RRT: Renal replacement therapy
SAPS: Simplified Acute Physiology Score
SF-36: Short Form Health Survey
SOFA: Sequential Organ Failure Assessment Score
SWI: Susceptibility-weighted imaging
TCD: Transcranial Doppler ultrasound
TISS: Therapeutic Intervention Scoring System
